# Low-Molecular-Weight Heparin Versus Aspirin in Early Management of Acute Ischemic Stroke: A Systematic Review and Meta-Analysis

**DOI:** 10.3389/fimmu.2022.823391

**Published:** 2022-02-24

**Authors:** Hui Xia, Ziyao Wang, Min Tian, Zunjing Liu, Zhenhua Zhou

**Affiliations:** ^1^ Graduate School of Beijing University of Chinese Medicine, Beijing, China; ^2^ Department of Neurology, China-Japan Friendship Hospital, Beijing, China; ^3^ Department of Neurology, Southwest Hospital, Third Military Medical University (Army Medical University), Chongqing, China

**Keywords:** ischemic stroke, stroke subtype, large-artery stenosis, low-molecular-weight heparin, aspirin

## Abstract

**Objectives:**

To evaluate the difference between low-molecular-weight heparin (LMWH) and aspirin in preventing early neurological deterioration (END) and recurrent ischemic stroke (RIS), post-recovery independence, and safety outcomes in acute ischemic stroke.

**Materials and Methods:**

We performed systematic searches of the PubMed, Embase, Web of Science, and Cochrane Library databases for full-text articles of randomized controlled trials (RCTs) of LMWH vs. aspirin in the early management of acute ischemic stroke. Information on study design, eligibility criteria, baseline information, and outcomes was extracted. Synthesized relative risks (RRs) with 95% confidence intervals (CIs) are used to present the differences between the two treatments based on fixed-effects models.

**Results:**

Five RCTs were retrieved from the online databases. The results showed no significant difference in efficacy outcomes between the two groups among unselected patients. Subgroup analysis showed that LMWH was significantly related to a lower incidence of END events [relative risk (RR): 0.44, 95% confidence interval (CI): 0.35–0.56] and reduced occurrence of RIS during treatment (OR: 0.34, 95% CI: 0.16–0.75) in non-cardioembolic stroke. LMWH significantly increased the number of patients with a modified Rankin scale (mRS) score of 0–1 at 6 months in patients with large-artery occlusive disease (LAOD) (RR: 0.50, 95% CI: 0.27–0.91). LMWH had a similar effect on symptomatic intracranial hemorrhage (sICH) and major extracranial hemorrhage during treatment to that of aspirin, except that LMWH was related to an increased likelihood of extracranial hemorrhage.

**Conclusions:**

In patients with acute non-cardioembolic ischemic stroke, especially that with large-artery stenosis, LMWH treatment significantly reduced the incidence of END and RIS, and improved the likelihood of independence (mRS 0–1) at 6 months compared with those with aspirin treatment. LMWH was related to an increased likelihood of extracranial hemorrhage among all patients; however, the difference in major extracranial hemorrhage and sICH was not significant. Choosing the appropriate patients and paying attention to the start time and duration of treatment are very important in the use of anticoagulation.

**Systematic Review Registration:**

http://www.crd.york.ac.uk/PROSPERO, identifier CRD42020185446.

## 1 Introduction

Early neurological deterioration (END) and recurrent ischemic stroke (RIS) are the most common conditions after acute ischemic stroke (AIS). END, defined as deterioration on the neurological scale (1), usually occurs within 24–72 hours from symptom onset and is often associated with a poor prognosis (2–4). Although antiplatelet agents (especially aspirin) are the most widely used and recommended medication in the early management of AIS, clinical neurologists often complain of their limited effect in halting symptom progression. END and RIS are still commonly observed after treatment with antiplatelet agents, meaning that not all patients benefit most from this therapy.

Despite being commonly used in clinical practice, the current guidelines do not recommend anticoagulant use in AIS (5). This conclusion was reached mainly based on two meta-analyses, which stated that anticoagulants were not associated with net short- or long-term benefits and had an increased bleeding risk (6, 7). However, we realized that the estimated effect may be subjective, as the results were primarily driven by one RCT, which accounted for approximately 80% of cases in all outcome analyses, and unfractionated heparin (UFH) was administered subcutaneously instead of routine intravenous injection; moreover, the incidence of bleeding due to anticoagulants was overestimated as placebo-controlled trials were involved. A meta-analysis comparing low-molecular-weight heparin (LMWH) with standard treatment aspirin has not been performed since 2002 (8). Recent randomized controlled trials (RCTs) have shown that direct oral anticoagulants (DOACs) could reduce ischemic lesion growth and improve recanalization with a similar risk of hemorrhagic transformation compared to that of aspirin (9, 10), indicating that anticoagulants may play a certain role in AIS. Heparin, a traditional and reliable anticoagulant, has also been found to have anti-inflammatory properties. Neuroinflammation is known to play an essential role in the pathophysiology of ischemic stroke, and these promising results prompted us to revisit the effects of LMWH in AIS as it has not been evaluated for many years.

In this meta-analysis, we aimed to provide more accurate estimates of the LMWH in the early management of AIS compared to aspirin. We paid special attention to END, as it has not been closely examined by other systematic reviews before.

## 2 Methods

We conducted this review in accordance to the PRISMA statements (64). See online **Supplementary Appendix Table 3** for the completed PRISMA checklist. Our review was registered with the International Prospective Register of Systematic Reviews (PROSPERO; http://www.crd.york.ac.uk/PROSPERO), registration number: CRD42020185446.

### 2.1 Search Strategy and Inclusion Criteria

We performed comprehensive searches in the PubMed, Embase, Web of Science, and Cochrane Library databases without language filter for full-text articles of RCTs of LMWH vs. aspirin in the early management of AIS from the inception of each database to October 1, 2020. The search was performed using the following terms: (stroke OR brain ischemia) AND (low-molecular-weight heparin) AND (antiplatelet or aspirin) AND randomized controlled trial. The inclusion criteria for the studies were as follows: (1) clinical diagnosis of AIS confirmed using computed tomography or magnetic resonance imaging, excluding the presence of intracerebral hemorrhage; (2) interventions were administered within 14 days of symptom onset; and (3) efficacy outcomes (END, RIS, and independence) and safety outcomes (including death and hemorrhagic adverse events) were reported. The exclusion criteria were as follows: (1) data could not be extracted, (2) single-arm studies, (3) participants received both anticoagulation and antiplatelet therapies, and (4) the allocation was not truly random or adequately concealed. The protocol for conducting and reporting this study was performed according to the items of the PRISMA methodology.

### 2.2 Outcome Assessment, Data Extraction, and Quality of Assessment

The outcomes assessed were END, RIS, short-and long-term independence (measured by mRS), death, and hemorrhagic adverse events. END is defined as an increase of 4 points or more in the National Institutes of Health Stroke Scale (NIHSS) score in most studies and could reflex functional change in neurological status (11). RIS is defined as any sudden and persistent deficit occurring >24 hours after the onset of the incident stroke, with both clinical and imaging findings of ischemic stroke diagnosed in an independent artery separated from index stroke territory (12). Hemorrhagic adverse events include sICH [defined as any CT-documented hemorrhage that was temporally related to deterioration in the patient’s clinical condition in the judgment of the clinical investigator (13)] and extracranial hemorrhage (such as gastrointestinal bleeding, hematoma, hematuria). A standardized, pre-piloted form was used to extract data, including the aforementioned outcomes, as well as study design, eligibility criteria, and baseline information, for the assessment of study quality and evidence synthesis. Two first authors (X.H and W.Z.) independently extracted and cross-checked the data. The Cochrane Collaboration’s tool was used to evaluate the methodological quality of eligible trials (random sequence generation, allocation concealment, blinding of participants and personnel, blinding of outcome assessment, incomplete outcome data, selective reporting, and other sources of bias) (14). The quality of each eligible study was assessed using RevMan version 5.3.5. Any discrepancy was resolved through discussion among all authors.

### 2.3 Statistical Analysis

STATA version 14.0 (Stata Corporation, College Station, TX, USA) was used to perform the meta-analysis. Synthesized relative risks (RRs) with 95% confidence intervals (CIs) are used to present the differences between the two treatments based on fixed-effects models (15). The chi-squared test and Higgins I^2^ statistics were used to evaluate the heterogeneity across studies (16, 17); *P*<0.01, and I^2^ >50% were considered to represent substantial heterogeneity. If there was no significance in heterogeneity degree, the fixed effect model (Mantel-Haenszel method) would be used. Otherwise, the random effect model (DerSimonian and Laird method) would be used. Sensitivity analysis was performed to assess how a single study affected the combined effect size. Subgroup analyses were performed according to stroke subtype, type of heparin, and National Institutes of Health Stroke Scale (NIHSS) score at baseline. Publication bias was evaluated using a funnel plot and quantified with Begg’s test and Egger’s test to assess funnel plot asymmetry (18).

## 3 Results

### 3.1 Search Results

**Figure 1:**
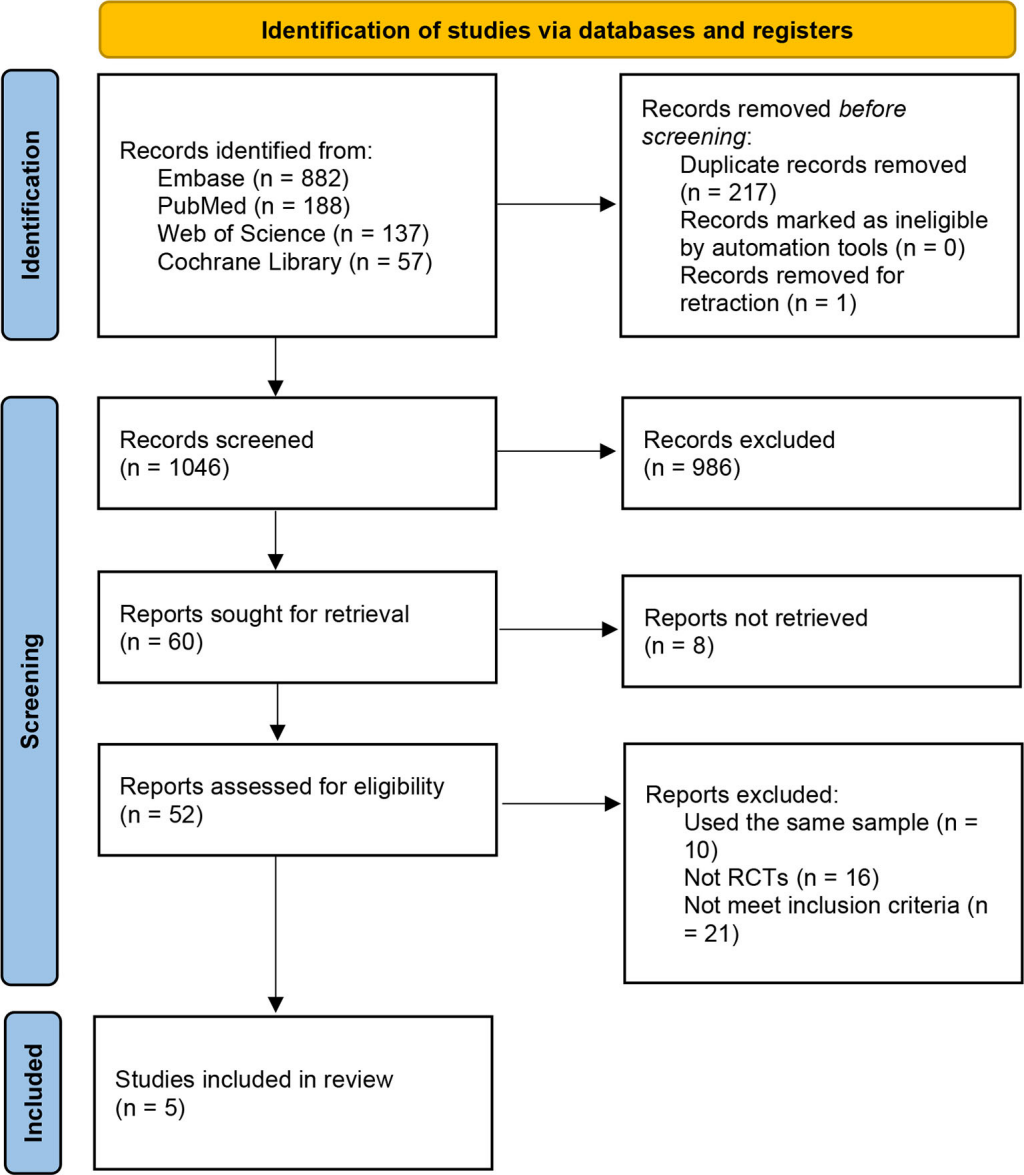
PRISMA flow diagram.

The search yielded a total of 932 relevant records. After screening, five RCTs met the inclusion criteria and were included in the analysis (**Figure 1**) (19–23). Two RCTs (Yi 2014 and Yi 2015) recruited patients from the same hospital over similar recruitment periods, and we confirmed that no patients were involved in both studies by contacting the corresponding author. Therefore, data from these two studies were analyzed separately.

### 3.2 Characteristics of Included RCTs

Five RCTs provided 4625 eligible cases for our analysis. For neurological assessment, three trials used the NHISS score, and two trials used the Scandinavian Stroke Scale (SSS) score. We converted SSS scores to NHISS scores using a previously developed conversion algorithm (24).

The characteristics of the included studies are listed in **Table 1**. For more details, please refer to the **Supplementary Appendix**.

**Table 1:** Characteristics of included studies and patients.

Study/year	Enrolled participants	Location	Stroke subtype	Heparin administration	Aspirin administration	Treatment period(d)	Median Age	Eligible/baseline NHISS score	Time onset to treatment	Follow up	Agent during follow-up	Outcome measurements
**HAEST/2000** (20)	449	Europe	All CE	Dalteparin	Oral 160 mg q.d.	14	80	NHISS ≤22/9^*^	21 h.	3 months	Oral anticoagulant	RIS, mRS 0–2, death, SICH, extracranial hemorrhage
				100 IU/kg s.c., b.i.d.								
**TAIST/2001** (19)	1484	Europe	24.7% CE	Tinzaparin	Oral 300 mg q.d.	10	74	NHISS ≥3/11^*^	24 h	6 months	Oral antithrombotic agents (antiplatelet or anticoagulant)	ND, mRS 0–2, death, extracranial hemorrhage, major extracranial hemorrhage
			32.6% AT	175 anti-Xa IU/kg or 100 anti-Xa IU/kg s.c. q.d								
			35.9% SAD									
			12.0% Other									
**FISS-tris/2007** (21)	353	Asia	All LAOD	Enoxaparin	Oral 160 mg q.d.	10	68	NHISS ≤22/6	29 h	6 months	Oral aspirin 80–300 mg q.d	ND, RIS, mRS 0–2, mRS 0–1, death, extracranial hemorrhage
				3800 anti-Xa IU/0–4 mL								
				s.c., b.i.d.								
**Yi/2014** (23)	1368	Asia	73% AT	Enoxaparin	Oral 200 mg q.d.	10	70^*^	NHISS ≤15/10	45 h	6 months	Oral aspirin 100 mg q.d.	ND, RIS, mRS 0–2, death, SICH, extracranial hemorrhage
			27% SAD	4000 anti-Xa IU/0–4 mL								
				s.c., b.i.d.								
**Yi/2015** (22)	969	Asia	69% AT	Enoxaparin	Oral 200mg q.d.	14	70^*^	NHISS ≤15/10	41 h	6 months	Oral aspirin 100 mg q.d.	ND, RIS, mRS 0–2, death, SICH, extracranial hemorrhage
			31% SAD,	4000 anti-Xa IU/0–4 mL								
				s.c., b.i.d.								

*represents data shown as the median score, otherwise data are shown as the mean score. CE, cardioembolism; LAOD, large artery occlusive disease; AT atherosclerosis; SAD, small artery disease; mRS, modified Rankin scale; NIHSS, National Institute of Health stroke scale; ND, neurological deterioration; SICH, symptomatic intracranial hemorrhage; q.d., once daily; b.i.d., twice daily; s.c., subcutaneous injection.

### 3.3 Risk of Bias of Included Studies

Details of the risk of bias assessment are available in **Supplementary Appendix Figures 1** and **2**. All five studies described the method of randomization and were considered low risk. The overall risk of bias in blinding, attrition, and selection was moderate. Owing to the small number of eligible studies, we did not explore publication bias.

### 3.4 Efficacy Outcomes

#### 3.4.1 END

Data were available for four trials, including 4174 participants. END was defined as a decrease of at least 5 points or a decrease of more than 2 points in the consciousness section of the SSS in TAIST 2001 (19), an increase of 4 points or more on NHISS or death at 10 days from baseline in FISS-tris 2007 (25), and an increase of 4 points or more on NHISS from day 2 to day 10 excluding those with hemorrhagic transformation or new infract in another vascular territory in Yi 2014 and Yi 2015 (22, 23). HAEST 2000 was excluded from this part of the analysis because END was not well reported (20). The results showed that LMWH significantly reduced END (RR: 0.57, 95% CI: 0.33–0.99). However, heterogeneity was significant (I^2^ = 91.9%, *P*<0.01) (**Supplementary Appendix Figure 3A**).

**Figure 2:**
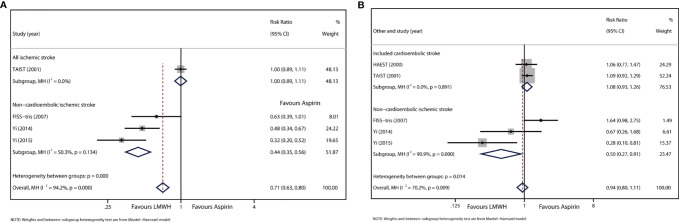
**(A)** Forest plot of the effects of LMWH vs. aspirin on the outcome of END. **(B)** Forest plot of the effects of LMWH vs. aspirin on the outcome of RIS.

A sensitivity analysis was performed by omitting the given study (**Supplementary Appendix Figure 3B**), and the results showed that the heterogeneity could be attributed to TAIST 2001. In this trial, all subtypes of ischemic stroke were included, and tinzaparin was administered once daily. This trial showed no difference in END occurrence between LMWH and aspirin (RR: 1.00, 95% CI: 0.89–1.11). We recalculated the effect size of the three remaining studies after excluding the main influence of heterogeneity. Heterogeneity decreased significantly, and no significant heterogeneity was detected. (I^2^ = 50.3%, *P*=0.134). Since the three trials excluded stroke caused by cardioembolism, we defined this group as the non-cardioembolism subgroup. In this subgroup, most strokes were large artery stenosis, and enoxaparin was administered twice daily. The results showed that LMWH was associated with a significant reduction in neurological deterioration relative to that with aspirin (RR: 0.44, 95% CI: 0.35–0.56) (**Figure 2A**).

#### 3.4.2 RIS During Treatment Period

Data were available for five trials involving 4625 patients. Data from TAIST 2001 included patients with uncertain recurrent stroke subtype. The treatment period of the included studies ranged from 10 to 14 days. Overall, there was no significant difference in RIS between LMWH and aspirin (RR: 1.02, 95% CI: 0.74–1.39), but significant heterogeneity was detected (I^2^ = 59.5%, *P*=0.042; **Supplementary Appendix Figure 4A**).

Subgroup analysis was performed to verify the disparity in stroke subtypes. For the subgroup analysis of FISS-tris 2007, Yi 2014, and Yi 2015 (**Figure 2B**), in which all patients were classified as having non-cardioembolic stroke, the result showed that RIS reduction was significantly associated with LMWH (RR: 0.50, 95% CI: 0.27–0.91). However, heterogeneity was still detected (I^2 =^ 90.9%, *P*<0.01). For another subgroup analysis of Yi 2014 and Yi 2015, in which the eligible baseline NHISS score was below 15 (**Supplementary Appendix Figure 4B**), the results showed that LMWH also significantly reduced RIS (RR: 0.40, 95% CI: 0.19–0.81), and no significant heterogeneity was found (I^2 =^ 37.7%, *P*=0.205). No other subgroup showed a difference in RIS between the LMWH and aspirin groups.

#### 3.4.3 Independence

Two trials (HAEST 2000 and FISS-tris 2007) reported an mRS score of 0–2 at the end of the treatment (10–14 days), including 802 patients. There was no significant difference in mRS score between LMWH- and aspirin-treated patients (RR: 1.03, 95% CI: 0.90–1.19), and heterogeneity was not detected (I^2^<0.01%, *P*=0.910) (**Supplementary Appendix Figure 5A**). All five RCTs reported an mRS score of 0–2 at the end of follow-up, which was 3 months for HAEST 2000, and 6 months for the other studies. This part of the analysis included 4623 participants and found no difference between LMWH and aspirin (RR: 1.00, 95% CI: 0.95–1.06). Heterogeneity was not detected (I^2^<0.01%, *P*=0.462; **Supplementary Appendix Figure 5B**). FISS-tris 2007 individually reported data of an mRS score of 0–1 at 3 months, which represented a full recovery from stroke. Data from 353 participants showed that LMWH significantly increased the proportion of patients with an mRS score of 0–1 (RR: 1.23, 95% CI: 1.00–1.51) (**Supplementary Appendix Figure 5C**).

### 3.5 Safety Outcomes

#### 3.5.1 Death From Any Cause During Treatment Period and at the End of Follow-up

All trials reported death during the treatment and follow-up periods. The results showed no significant difference in mortality between LMWH and aspirin both in the treatment period (RR: 1.14, 95% CI: 0.97–1.27) and at the end of follow-up (RR: 1.01, 95% CI: 0.92–1.10). No significant heterogeneity was detected (I^2^<0.01%; **Supplementary Appendix Figure 6**).

#### 3.5.2 Symptomatic Intracranial Hemorrhage During the Treatment Period

**Figure 3:**
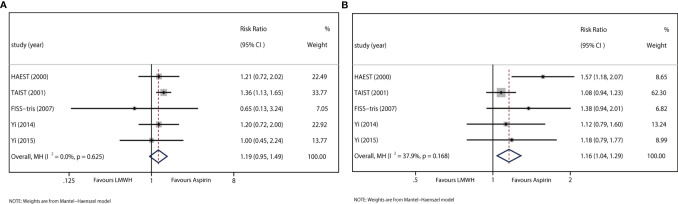
**(A)** Forest plot of the effects of LMWH vs. aspirin on the outcome of sICH. **(B)** Forest plot of the effects of LMWH vs. aspirin on the outcome of extracranial hemorrhage.

All trials reported data on sICH. The results showed no difference between LMWH and aspirin regarding sICH (RR: 1.19, 95% CI: 0.95–1.49). Heterogeneity was not detected between study results (I^2^<0.01%, *P*=0.625) (**Figure 3A**).

#### 3.5.3 Extracranial Hemorrhage During the Treatment Period

All trials recorded data on extracranial hemorrhage during the treatment period. The results indicated that LMWH was significantly associated with extracranial hemorrhage (RR: 1.16, 95% CI: 1.04–1.29; I^2 =^ 37.9%, *P*=0.168) (**Figure 3B**).

Major extracranial hemorrhage, defined as any fatal bleeding or bleeding severe enough to require transfusion or operation, was reported in TAIST 2001. The results indicated that LWMH was not significantly associated with major extracranial hemorrhage (RR: 1.12, 95% CI: 0.75–1.67).

## 4 Discussion

The efficacy and safety of aspirin in AIS have been validated in large trials (26, 27); however, some patients still develop END or RIS after receiving antiplatelet therapy. Therefore, clinical neurologists consider anticoagulants to be a feasible therapy. Pharmacological studies have shown that heparin can reduce blood viscosity, promote fibrinolysis, prevent the growth of early ischemic lesions, and have better anti-inflammatory effects than aspirin in AIS (28–31). Although previous meta-analyses have provided no recommendation on anticoagulants in AIS, the conclusion may be subject to imprecision and no evaluation based on stroke etiology has been performed.

In our meta-analysis, the results showed no significant differences in short- or long-term efficacy between LMWH and aspirin among all patients, which is consistent with previous studies. However, in patients with non-cardioembolic stroke, a significant difference in short-term efficacy between LMWH and aspirin was observed. LMWH was associated with a significant reduction in END (RR: 0.44, 95% CI: 0.35–0.56; absolute risk reduction [ARR]: 9%, 95% CI: 7.94–10.06%) and RIS (RR: 0.50, 95% CI: 0.27–0.91; ARR: 1.19%, 95% CI: 0.79–1.59%). To the best of our knowledge, this is the first meta-analysis to evaluate the estimated effect of heparin in the early management of AIS with consideration of stroke etiology. The positive results were mainly driven by three studies (21–23), in which patients shared similar characteristics (non-cardioembolic stroke and most were diagnosed with large-artery stenosis), and LMWH was administered twice daily. As an important factor of poor prognosis (32), END has been clinically observed to be relevant to atherosclerotic diseases such as atherosclerosis (AT) and small artery disease (SAD) (33, 34). This explains the higher incidence of END in these three trials since all strokes were caused by large-artery occlusive disease (LAOD) in FISS-tris, and patients were diagnosed with either AT or SAD in Yi 2014/2015. Few studies have revealed the intrinsic link between END and stroke etiology. The most common reason for END could be attributed to ischemic lesion growth (35), and heparin might prevent END by reducing ischemic extension and salvage the ischemic penumbra (36). Complete or partial lysis of intraluminal thrombus has been observed in patients who received intravenous heparin (37), indicating that anticoagulants are capable of promoting innate thrombolysis and improving blood flow in the ischemic area. However, this effect could be limited in cardioembolic stroke, which is caused by emboli originating from an organized heparin-unresponsive thrombus within the heart (20). It is noteworthy that although FISS-tris showed no reduction in RIS in the LMWH group, this is possibly due to chance, as the incidence of RIS in patients receiving aspirin was significantly lower in FISS-tris than in other larger RCTs (0% vs. 3%) (21, 26).

The mRS is a simplified overall assessment of disability, with scores ranging from 0 (no symptoms at all) to 6 (death), with 5 indicating severe disability (complete dependency). A functional outcome of independence as reflected by an mRS score of 0–2 was similar between LMWH- and aspirin-treated patients, which has been interpreted as heparin and aspirin sharing equal efficacy in improving patients’ disability (6). However, we realized that LMWH might enable more patients to achieve full recovery than aspirin, as one of our included trials (FISS-tris) suggested that LMWH was significantly associated with a higher likelihood of an mRS of 0–1 in patients with LAOD at 6 months. An mRS of 0–1 was usually considered as a favorable outcome in previous AIS thrombolysis trials (38, 39), and differences in the dichotomization of mRS strongly influenced the interpretation of the results; for example, although the difference in the number of patients with an mRS score of 0–2 was not significant, there was a significant difference in the number of patients with an mRS of 0–1 between the alteplase and placebo groups in ECASS III, supporting the use of thrombolysis therapy in AIS after 3–4.5 hours (recommended by current guideline) (40). Similarly, the significant association between an mRS score of 0–1 and LMWH indicates that LMWH is effective in improving 6-month independence in AIS patients with LAOD. According to the discussion above, patients with LAOD could benefit from LMWH in the short term, and the long-term efficacy of LMWH in patients with AT has also been proven by TOAST (1) (although LMWH was administered differently), strongly supporting that LMWH is capable of improving functional outcomes in stroke patients with large-artery stenosis.

We did not observe differences in the number of deaths at any time point, indicating that LMWH is relatively safe in the early management of AIS relative to aspirin. Although the incidence of extracranial hemorrhage increased significantly in patients who received LMWH, as most mild bleeding is reversible, major or severe bleeding is the main clinical concern. There was no significant difference in sICH or major extracranial hemorrhage between the LMWH and aspirin groups. Overall, in every 1000 patients who received LMWH rather than aspirin, 55 ENDs and nine RISs would be prevented; at the same time, 19 cases of extracranial hemorrhage and three cases of SICH would occur. As all complications mentioned above were “symptomatic”, we concluded that the net benefit of LMWH was observable. Furthermore, the benefits of LMWH were mainly seen in non-cardioembolic patients, and the trial (HAEST) that included only patients with cardioembolism found no difference either in RIS or independence (20). According to the consensus in the ESC Guidelines, patients with atrial fibrillation and moderate stroke should initiate or continue anticoagulation 6 days after stroke onset (41). Thus, whether the administration of LMWH in cardioembolic patients should be postponed requires further investigation.

There has been increasing evidence over the past decade that peripheral innate and adaptive immune cells, such as neutrophils and natural killer cells, may play an important role in the pathophysiology of ischemic stroke. The influx of neutrophils may cause increased vascular resistance due to increased blood viscosity and cellular obstruction, leading to cerebral infarction associated with collateral failure (42–44). A recent study showed that toll-like receptors (TLRs) are involved in the activation of inflammatory responses during the acute phase after ischemic stroke (45, 46). TLR-2 and -4 persist for at least 7 days after reperfusion, promoting exacerbation of acute inflammation and impeding neurological recovery (45) Histones released by dead cells can induce thrombin generation by activating platelets *via* TLR-2 and TLR-4 (47), which may lead to immunothrombosis after stroke (48). Heparin could lower TLR-4 protein expression and prevent histone interactions with platelets, which has been shown to reduce the risk of immunothrombosis (49). At the same time, secondary neuroinflammation in AIS leads to compromised integrity of the blood-brain barrier (BBB), allowing water molecules and blood components to enter the extracellular space of the brain, resulting in serious clinical consequences (50–53). The extent of BBB is associated with stroke severity and progression (54, 55). Thus, preventing BBB disruption is considered a potential therapeutic strategy for improving END. Glycocalyx, a polysaccharide protein complex that covers the surface of vascular endothelial cells, is abundantly expressed on the endothelial cells of the BBB and has been shown to regulate BBB permeability (51). It plays a key role in the inflammatory process by interrupting the cycle of endothelial dysfunction and inflammation (56). After stroke, endothelial cells are exposed to neuroinflammation and elicit degradation of the glycocalyx (57–59). In atherosclerosis, one of the main pathogeneses of stroke, glycocalyx degradation promotes lipid deposition in the vessel walls and reduces endothelial cell expression of endothelial nitric oxide synthase (eNOS), causing loss of vasodilation (60). LWMH could suppress glycocalyx shedding in a dose-dependent manner as an inhibitor of heparanase activity (61, 62), thereby improving BBB leakage, brain edema, decreasing the expression of inflammatory factors, and improving neurologic outcomes (63).

The strengths of our study were that the meta-analysis was conducted and reported according to the PRISMA methodology; the risk of bias of each included study was carefully screened using the Cochrane Collaboration’s tool, finding that all trials were adequately randomized and accessor-blinded; and we analyzed the heterogeneity between trials based on stroke etiology, which provides information on how to achieve precision treatment. The limitations were that we did not obtain original data for each trial; hence, no evaluation based on the individual participant level was performed, and two trials might be not sufficiently normative as they were not registered on NCT or ISRCTN (the methodology of two trials was considered appropriate) (22, 23). In addition, our positive results are mainly based on three Asian studies; as racial differences in stroke etiology and pathogenesis are essential factors underlying drug selection, the risk of bias cannot be neglected. Furthermore, only one RCT study used the mRS score of 0-1 to evaluate the effect of LMWH on cerebral ischemic injury, which could not provide the enough evidence to draw the conclusion. However, mRS score of 0-1 is an important measurement in clinical practice, we emphasized this score in our article. It is promising that LMWH benefits certain patients with non-cardioembolic stroke. We believe that further basic and clinical studies should be conducted on the application of LMWH to elucidate the population most likely to benefit and reveal the mechanisms of LMWH in the treatment of AIS.

## 5 Conclusion

In patients with acute non-cardioembolic ischemic stroke, especially those with large-artery stenosis, LMWH significantly reduced the incidence of END and RIS, and improved independence (mRS 0–1) at 6 months relative to those with aspirin. We found no significant difference between LMWH and aspirin in improving patients’ long-term functional outcomes measured as reflected by an mRS score of 0–2. LMWH treatments was related to increased risk of extracranial hemorrhage among all patients; however, the difference in major extracranial hemorrhage and sICH was not significant. LMWH was associated with a net benefit in non-cardioembolic stroke. The current situation is that anticoagulation therapy has not been recommended for non-cardioembolic ischemic stroke; and the increased risk for bleeding, instead of not reducing the risk of neurological deterioration and recurrent ischemic stroke in early acute ischemic stroke, is the main reason. Therefore, choosing the appropriate patients and paying attention to the start time and duration of treatment are very important. Nowadays, lots of physician still use the individualized LMWH in the treatment of noncardiogenic acute ischemic stroke. Hope our study can trigger more experts to re-recognize.

## Data Availability Statement

The original contributions presented in the study are included in the article/**Supplementary Material**. Further inquiries can be directed to the corresponding authors.

## Author Contributions

ZL, ZW, and HX designed the systematic review. ZW, HX, and MT performed the literature search the literature search, reviewed all publications, extracted the information and data from the included studies, performed the data analysis, and produced the figures and tables. ZW, HX, and MT wrote the manuscript. ZL and ZZ revised the manuscript. All authors have read and agreed to the published version of the manuscript.

## Funding

This study was supported by grants from National Natural Science Foundation of China (No. 52073310) and Elite Medical Professionals project of China-Japan Friendship Hospital (NO. ZRJY2021-BJ03) and Technical innovation project in major clinical fields (CX2019LC103).

## Conflict of Interest

The authors declare that the research was conducted in the absence of any commercial or financial relationships that could be construed as a potential conflict of interest.

## Publisher’s Note

All claims expressed in this article are solely those of the authors and do not necessarily represent those of their affiliated organizations, or those of the publisher, the editors and the reviewers. Any product that may be evaluated in this article, or claim that may be made by its manufacturer, is not guaranteed or endorsed by the publisher.
