# Corrigendum: Low-molecular-weight heparin versus aspirin in early management of acute ischemic stroke: A systematic review and meta-analysis

**DOI:** 10.3389/fimmu.2022.1052766

**Published:** 2022-11-10

**Authors:** Hui Xia, Ziyao Wang, Min Tian, Zunjing Liu, Zhenhua Zhou

**Affiliations:** ^1^ Graduate School of Beijing University of Chinese Medicine, Beijing, China; ^2^ Department of Neurology, China-Japan Friendship Hospital, Beijing, China; ^3^ Department of Neurology, Southwest Hospital, Third Military Medical University (Army Medical University), Chongqing, China

**Keywords:** ischemic stroke, stroke subtype, large-artery stenosis, low-molecular-weight heparin, aspirin

In the published article, there was an error regarding the affiliations for Hui Xia and Ziyao Wang. As well as having the affiliation “Department of Neurology, China-Japan Friendship Hospital, Beijing, China”, they should also have “Graduate School of Beijing University of Chinese Medicine, Beijing, China”.

The authors apologize for this error and state that this does not change the scientific conclusions of the article in any way. The original article has been updated.

## Publisher’s note

All claims expressed in this article are solely those of the authors and do not necessarily represent those of their affiliated organizations, or those of the publisher, the editors and the reviewers. Any product that may be evaluated in this article, or claim that may be made by its manufacturer, is not guaranteed or endorsed by the publisher.

