# Supplementary material for: Low-Molecular-Weight Heparin Versus Aspirin in Early Management of Acute Ischemic Stroke: A Systematic Review and Meta-Analysis
Source: Front Immunol. 2022 Feb 24;13:823391. doi: 10.3389/fimmu.2022.823391 (PMC8908308; doi:10.3389/fimmu.2022.823391)
Supplement: Supplementary file 1 [file DataSheet_1.docx]

Supplementary Material

# Supplementary Data

## Literature Search Details

Ovid EMBASE(R) from the inception to present – Search ran 1/21/2021

1. 'brain ischemia'/exp OR 'brain ischemia' OR stroke:ab,ti OR 'cerebrovascular accident':ab,ti OR apoplexy:ab,ti OR 'brain infarction':ab,ti

2. 'low molecular weight heparin'/exp OR lmwh:ab,ti OR nadroparin:ab,ti OR fraxiparin:ab,ti OR danaproid:ab,ti OR 'enoxaparin':ab,ti OR 'dalteparin':ab,ti OR 'tinzaparin':ab,ti OR 'fragmin':ab,ti

3. 'antithrombocytic agent'/exp OR aspirin:ab,ti OR cilostazol:ab,ti OR clopidogrel:ab,ti OR dipyridamole:ab,ti OR ticlopidine:ab,ti OR prasugrel:ab,ti OR 'glycoprotein iib/iiia receptor antagonists':ab,ti

4. 'randomized controlled trial'/exp OR 'controlled clinical trial':ab,ti OR randomized:ab,ti OR placebo:ab,ti OR randomly:ab,ti OR trial:ab,ti OR groups:ab,ti

5. 1 AND 2 AND 3 AND 4

No language filter was applied. The first search was conducted in Ovid EMBASE. Subject headings and keywords were adapted for the other databases.

## Details of the risk of bias assessment

The sealed envelope randomization was used in FISS-tris 2007, Yi 2014, and Yi 2015, and the randomization schedule of HAEST 2000 was computer-generated (SAS, version 6.10). TAIST 2001 reported the allocation was randomized but the generation of the random allocation sequence was not clearly described. Allocation concealment was adequate for all 5 trials.

Adequate blinding is relatively important in clinical trials of acute stroke, to reduce the bias in detecting recurrent ischemic stroke, symptomatic intracranial hemorrhage, etc. HAEST 2000 and TAIST 2001 were double-blinded trials and accessors were also unaware of treatment allocation. FISS-tris 2007 and Yi 2014 were reported as trials with blinded outcome assessment; however, the blinding of participants and personnel was unclear. Accessors were also blinded in Yi 2015, but the patients and personnel haven't been blinded, leading to a relatively high risk of bias in performance.

A total number of 58 patients were lost during follow-up. HAEST 2000 and Yi 2014 trail stated clearly there were no lost in follow-up by flow diagrams, so the risk of attrition bias was considered low, while the risk of other trials remains unknown. The original protocols were available for no trials so the risk of selective reporting bias could not be estimated. Some data inconsistency was found in Yi 2014.

## Characteristics of Included RCTs

Of the included 5 RCT, 3 were conducted in Asia, and 2 were in Europe, involving 4625 subjects. All trials excluded patients with intracranial hemorrhage on baseline CT scan and restricted patient's blood pressure within 220/120 mmHg. All patients in these RCTs were enrolled within 48 hours from symptoms onset. All trials were conducted with low-molecular-weight heparin (LMWH) and aspirin, most of the patients received treatment within 24 hours, and the treatment last for 10-14 days.

Enoxaparin (3800 IU to 4000 IU twice daily) was used in three trials (FISS-tris 2007, Yi 2014, and Yi 2015). Dalteparin was used in one trial, HAEST 2000, with a weight-adjusted injection, 100 IU/kg twice daily. TAIST 2001 was conducted with Tinzaparin, also administrated with weight-adjusted injection, 175 IU/kg or 100 IU/kg, but only once daily. All LMWH was given through subcutaneous injection. The dose of oral aspirin in control groups ranged from 160 mg to 300 mg in included trials and was given once daily. As for the selection of stroke subtype, HAEST 2000 included patients only with atrial fibrillation and stroke due to cardioembolism. FISS-tris 2007, Yi 2014 and Yi 2015 excluded patients with cardioembolism and most of the patients diagnosed with large artery stenosis. TAIST 2001 didn’t screen for specific stroke subtype.

## Publication Bias

Owing to the small number of eligible studies, we did not explore publication bias.

# Supplementary Figures and Tables

## Supplementary Figures


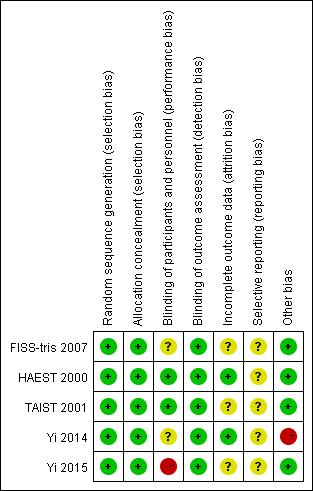


**Supplementary Figure 1.** Risk of bias graph review each


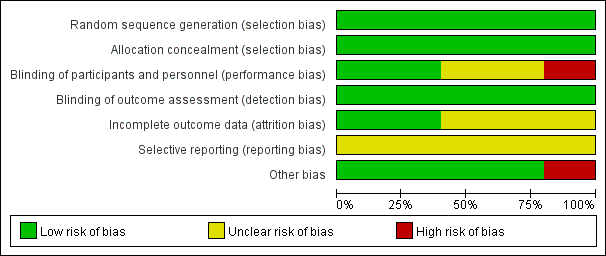


**Supplementary Figure 2.** Risk of bias graph summary


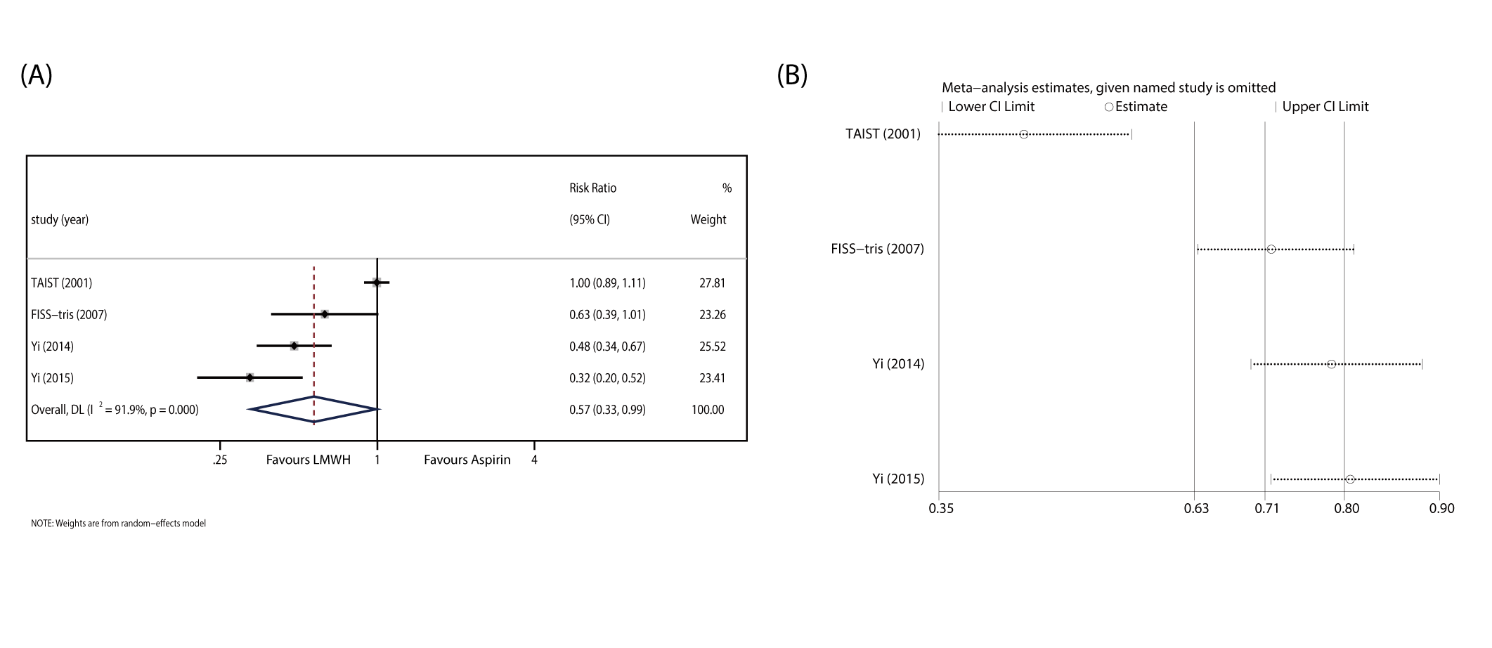


**Supplementary figure 3.** (A) Forest plot of the effects of LMWH versus aspirin on the outcome of END. (B) Sensitivity analysis, given study was omitted.


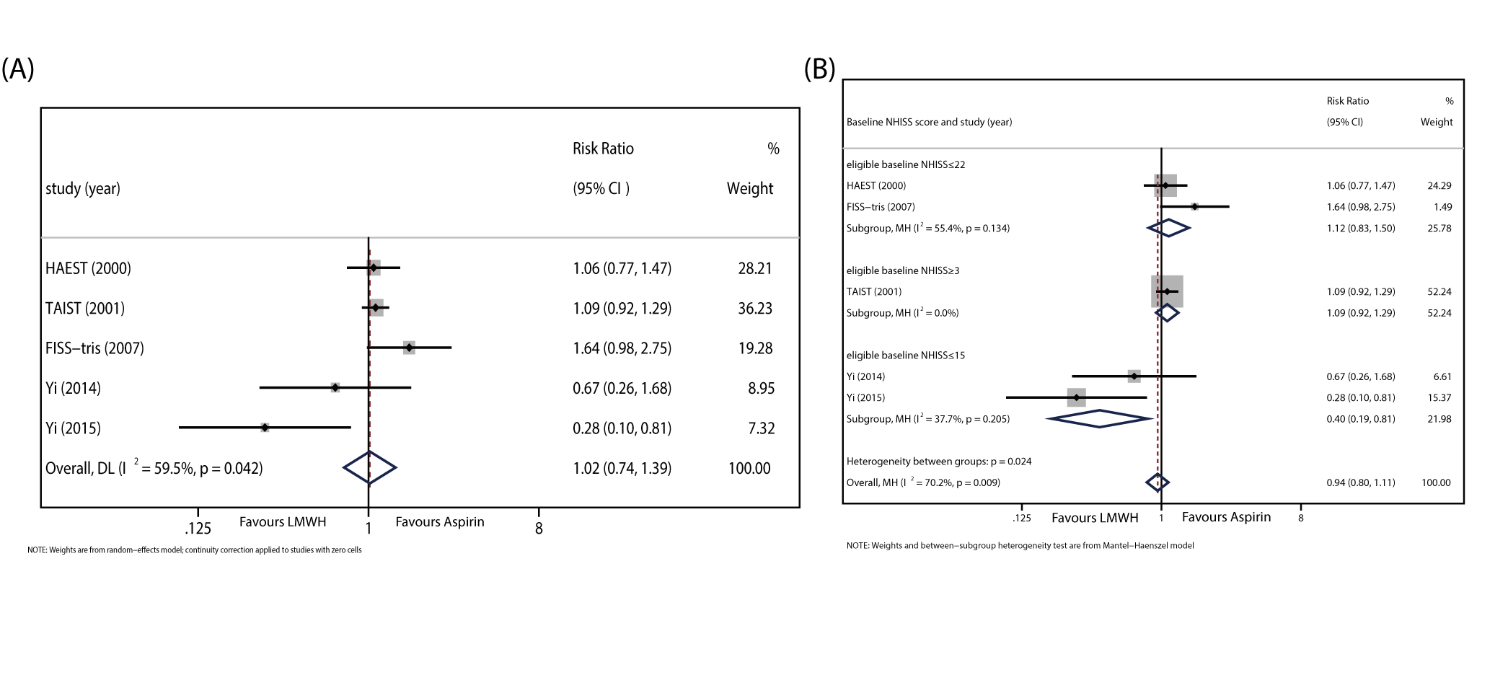


**Supplementary figure 4.** (A) Forest plot of the effects of LMWH versus aspirin on the outcome of RIS. (B) Subgroup analysis based on eligible NHISS score at baseline.


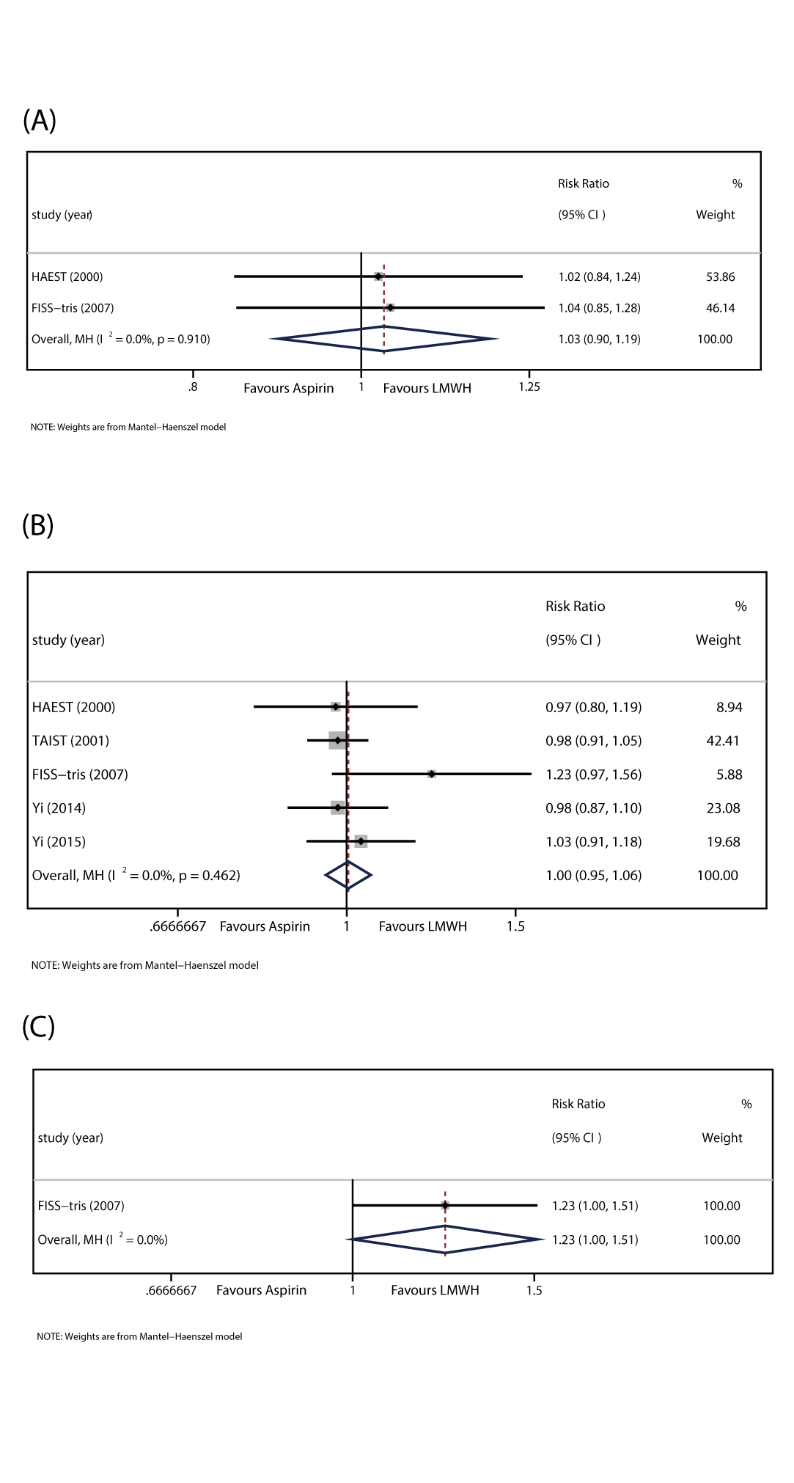


**Supplementary figure 5.** (A) Forest plot of the effects of LMWH versus aspirin on mRS 0-2 at the end of treatment. (B) Forest plot of the effects of LMWH versus aspirin on mRS 0-2 at the end of follow-up. (C) Forest plot of the effects of LMWH versus aspirin on mRS 0-1 at the end of follow-up.


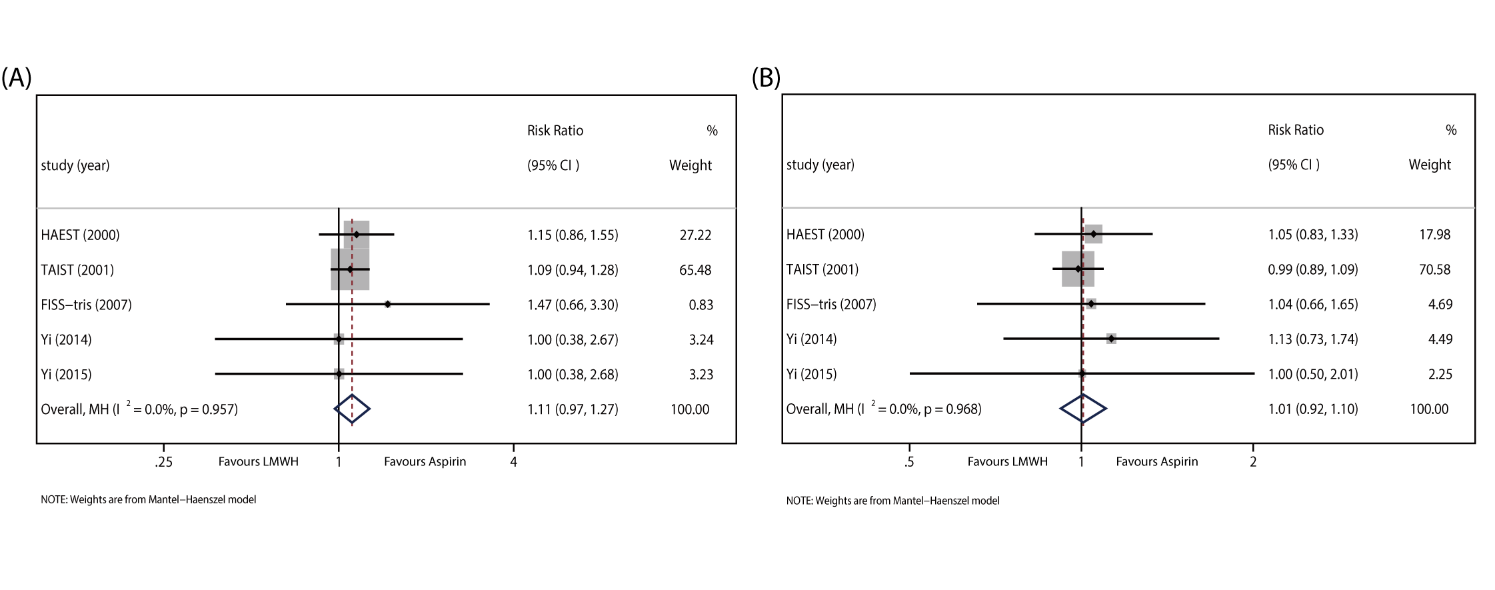


**Supplementary figure 6.** (A) Forest plot of the effects of LMWH versus aspirin on the outcome of death at the end of treatment. (B) Forest plot of the effects of LMWH versus aspirin on the outcome of death at the end of follow-up.

## Supplementary Tables

| Study/ year | **LMWH** | | | | | | **Aspirin** | | | | | |
| --- | --- | --- | --- | --- | --- | --- | --- | --- | --- | --- | --- | --- |
|  | ***Enrolled Participants*** | ***END (%)*** | ***RIS During Treatment Period (%)*** | ***Good Outcome At The End Of Treatment Period (%)*** | ***Good Outcome At The End Of Follow-Up (%)*** | ***Very***  ***Good***  ***Outcome***  ***At The***  ***End Of***  ***Follow-Up (%)*** | ***Enrolled Participants*** | ***END (%)*** | ***RIS During Treatment Period (%)*** | ***Good Outcome At The End Of Treatment Period (%)*** | ***Good Outcome At The End Of Follow-Up (%)*** | ***Very***  ***Good***  ***Outcome***  ***At The***  ***End Of***  ***Follow-Up (%)*** |
| HAEST/  2000 | 224 | NA | 19(8.5) | 83(37.1) | 76(33.9) | NA | 225 | NA | 17(7.5) | 81(36) | 79(35.1) | NA |
| TAIST/  2001 | 995 | 116(11.7) | 40^*^ | NA | 400(40.2) | NA | 491 | 58(11.8) | 15^*^ | NA | 205(41.8) | NA |
| FISS-tris/  2007 | 180 | 12(6.7) | 2(1.1) | 92(51.1) | 131(72.8) | 98(54.4) | 173 | 24(13.8) | 0(0) | 85(49.1) | 111(64.2) | 76(43.9) |
| Yi/  2014 | 683 | 27(3.9) | 3(0.4) | NA | 506(74.1) | NA | 685 | 81(11.8) | 6(0.8) | NA | 513(74.9) | NA |
| Yi/  2015 | 483 | 15(3.1) | 3(0.6) | NA | 299(61.9) | NA | 486 | 72(14.8) | 18(3.7) | NA | 293(60.3) | NA |
| All | 2565 | 170(7.3) | 27(1.7) | 175(43.3) | 1412(55) | 98(54.4) | 2060 | 235(12.8) | 41(2.6) | 166(41.7) | 1201(58.3) | 76(43.9) |

^*^Includes patients where recurrent stroke subtype was uncertain.

**Supplementary table 1.** Efficacy outcomes reported in each trial

| Study/ year | **LMWH** | | | | | | **Aspirin** | | | | | |
| --- | --- | --- | --- | --- | --- | --- | --- | --- | --- | --- | --- | --- |
|  | ***Enrolled Participants*** | ***Death From Any Cause During Treatment Period (%)*** | ***Death From Any Cause***  ***At The End Of Follow-Up (%)*** | ***Symptomatic Intracranial Hemorrhage During The Treatment Period (%)*** | ***Extracranial***  ***Hemorrhage During The Treatment Period (%)*** | ***Major Extracranial Hemorrhage During The Treatment Period (%)*** | ***Enrolled Participants*** | ***Death From Any Cause During Treatment Period (%)*** | ***Death From Any Cause***  ***At The End Of Follow-Up (%)*** | ***Symptomatic Intracranial Hemorrhage During The Treatment Period (%)*** | ***Extracranial Hemorrhage During The Treatment Period (%)*** | ***Major Extracranial Hemorrhage During The Treatment Period (%)*** |
| HAEST/  2000 | 224 | 21(9.3) | 40(17.9) | 6(2.7) | 13(5.9) | NA | 225 | 16(7.1) | 37(16.4) | 4(1.8) | 4（1.8） | NA |
| TAIST/  2001 | 995 | 46(4.6) | 143(14.4) | 10(1) | 66(6.6) | 4(0.4) | 491 | 17（3.5） | 73（14.9） | 1(0.2) | 26(5.3) | 2(0.4) |
| FISS-tris/  2007 | 180 | 1(0.1) | 9(5) | 1(0.6) | 9(5) | NA | 173 | 0(0) | 8(4.6) | 2(1.6) | 4(2.3) | NA |
| Yi/  2014 | 683 | 2(0.3) | 9(1.3) | 6(0.8) | 14(2) | NA | 685 | 2(0.3) | 7(1) | 4(0.5) | 11(1.6) | NA |
| Yi/  2015 | 483 | 2(0.4) | 4(0.8) | 3(0.6) | 10(2) | NA | 486 | 2(0.4) | 4(0.8) | 3(0.6) | 7(1.4) | NA |
| All | 2565 | 72(2.8) | 205(8) | 26(1) | 112(4.4) | 4(0.4) | 2060 | 37(1.8) | 129(6.3) | 14(0.7) | 52(2.5) | 2(0.4) |

**Supplementary table 2.** Adverse events reported in each trial.

| **Section/topic** | **#** | **Checklist item** | **Reported on page #** |
| --- | --- | --- | --- |
| **TITLE** | | |  |
| Title | 1 | Identify the report as a systematic review, meta-analysis, or both. | 1 |
| **ABSTRACT** | | |  |
| Structured summary | 2 | Provide a structured summary including, as applicable: background; objectives; data sources; study eligibility criteria, participants, and interventions; study appraisal and synthesis methods; results; limitations; conclusions and implications of key findings; systematic review registration number. | 2 |
| **INTRODUCTION** | | |  |
| Rationale | 3 | Describe the rationale for the review in the context of what is already known. | 2 |
| Objectives | 4 | Provide an explicit statement of questions being addressed with reference to participants, interventions, comparisons, outcomes, and study design (PICOS). | 2 |
| **METHODS** | | |  |
| Protocol and registration | 5 | Indicate if a review protocol exists, if and where it can be accessed (e.g., Web address), and, if available, provide registration information including registration number. | 3 |
| Eligibility criteria | 6 | Specify study characteristics (e.g., PICOS, length of follow-up) and report characteristics (e.g., years considered, language, publication status) used as criteria for eligibility, giving rationale. | 3 |
| Information sources | 7 | Describe all information sources (e.g., databases with dates of coverage, contact with study authors to identify additional studies) in the search and date last searched. | 3 |
| Search | 8 | Present full electronic search strategy for at least one database, including any limits used, such that it could be repeated. | 3 |
| Study selection | 9 | State the process for selecting studies (i.e., screening, eligibility, included in systematic review, and, if applicable, included in the meta-analysis). | 3 |
| Data collection process | 10 | Describe method of data extraction from reports (e.g., piloted forms, independently, in duplicate) and any processes for obtaining and confirming data from investigators. | 3 |
| Data items | 11 | List and define all variables for which data were sought (e.g., PICOS, funding sources) and any assumptions and simplifications made. | 3 |
| Risk of bias in individual studies | 12 | Describe methods used for assessing risk of bias of individual studies (including specification of whether this was done at the study or outcome level), and how this information is to be used in any data synthesis. | 3 |
| Summary measures | 13 | State the principal summary measures (e.g., risk ratio, difference in means). | 3 |
| Synthesis of results | 14 | Describe the methods of handling data and combining results of studies, if done, including measures of consistency (e.g., I^2^) for each meta-analysis. | 3 |

Page 1 of 2

| **Section/topic** | **#** | **Checklist item** | **Reported on page #** |
| --- | --- | --- | --- |
| Risk of bias across studies | 15 | Specify any assessment of risk of bias that may affect the cumulative evidence (e.g., publication bias, selective reporting within studies). | 3 |
| Additional analyses | 16 | Describe methods of additional analyses (e.g., sensitivity or subgroup analyses, meta-regression), if done, indicating which were pre-specified. | 3 |
| **RESULTS** | | |  |
| Study selection | 17 | Give numbers of studies screened, assessed for eligibility, and included in the review, with reasons for exclusions at each stage, ideally with a flow diagram. | 4-6 |
| Study characteristics | 18 | For each study, present characteristics for which data were extracted (e.g., study size, PICOS, follow-up period) and provide the citations. | 4-6 |
| Risk of bias within studies | 19 | Present data on risk of bias of each study and, if available, any outcome level assessment (see item 12). | 4-6 |
| Results of individual studies | 20 | For all outcomes considered (benefits or harms), present, for each study: (a) simple summary data for each intervention group (b) effect estimates and confidence intervals, ideally with a forest plot. | 4-6 |
| Synthesis of results | 21 | Present results of each meta-analysis done, including confidence intervals and measures of consistency. | 4-6 |
| Risk of bias across studies | 22 | Present results of any assessment of risk of bias across studies (see Item 15). | 4-6 |
| Additional analysis | 23 | Give results of additional analyses, if done (e.g., sensitivity or subgroup analyses, meta-regression [see Item 16]). | 4-6 |
| **DISCUSSION** | | |  |
| Summary of evidence | 24 | Summarize the main findings including the strength of evidence for each main outcome; consider their relevance to key groups (e.g., healthcare providers, users, and policy makers). | 7-9 |
| Limitations | 25 | Discuss limitations at study and outcome level (e.g., risk of bias), and at review-level (e.g., incomplete retrieval of identified research, reporting bias). | 7-9 |
| Conclusions | 26 | Provide a general interpretation of the results in the context of other evidence, and implications for future research. | 7-9 |
| **FUNDING** | | |  |
| Funding | 27 | Describe sources of funding for the systematic review and other support (e.g., supply of data); role of funders for the systematic review. | 10 |

*From:*  Moher D, Liberati A, Tetzlaff J, Altman DG, The PRISMA Group (2009). Preferred Reporting Items for Systematic Reviews and Meta-Analyses: The PRISMA Statement. PLoS Med 6(7): e1000097. doi:10.1371/journal.pmed1000097

For more information, visit: **www.prisma-statement.org**.

**Supplementary table 3.** PRISMA checklist.
